# 632. Cytomegalovirus Infections in Patients with Relapsed/Refractory Multiple Myeloma on Novel T-Cell Redirecting Therapy

**DOI:** 10.1093/ofid/ofae631.197

**Published:** 2025-01-29

**Authors:** Jennifer Makhoul, Silpa Jetty, Fareed Khawaja, Krina Patel, Hans C Lee, Sandra Horowitz, Amy Spallone, Ella Ariza Heredia, Roy F Chemaly

**Affiliations:** University of Texas Health Science Center at Houston/MD Anderson Cancer Center, Houston, TX; University of Texas Health Science Center at Houston/MD Anderson Cancer Center, Houston, TX; The University of Texas MD Anderson Cancer Center, Houston, Texas; MD Anderson Cancer Center, Houston, Texas; The University of Texas MD Anderson Cancer Center, Houston, Texas; UT MD Anderson Cancer Center, Houston, Texas; University of Texas MD Anderson Cancer Center, Houston, Texas; The University of Texas MD Anderson Cancer Center, Houston, Texas; University of Texas MD Anderson Cancer Center, Houston, Texas

## Abstract

**Background:**

The advent of T-cell redirecting therapies such as chimeric antigen receptor (CAR) T cells and bispecific antibodies (BsAbs) has reshaped the treatment of relapsed/refractory multiple myeloma (R/R MM), but their immunomodulatory effects potentially increase the risk of infections, including cytomegalovirus (CMV). We compared the rate of CMV viremia, clinically significant CMV infection (CS-CMVi), CMV end organ disease, and CMV related mortality in R/R MM patients receiving BsAbs, CAR T cells or non-T-cell redirecting standard of care (SOC) treatment.

Patients’ characteristics

**Figure:**
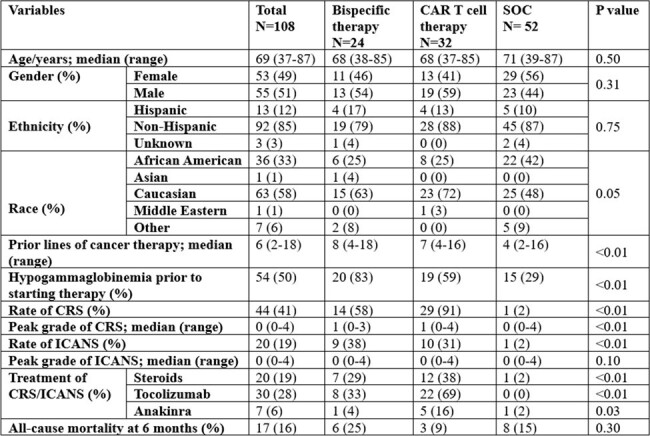

Abbreviations:

SOC: standard of care; CRS: cytokine release syndrome; ICANS: immune effector cell-associated neurotoxicity syndrome

**Methods:**

We conducted a retrospective cohort study in patients with R/R MM who underwent treatment with BsAbs, CAR-T cell or SOC regimens from January 2023 to June 2023. Patient demographics, oncologic history, complications related to therapy as well as CMV characteristics and outcomes were collected. The primary outcomes of interest included CMV viremia, CS-CMVi, CMV end organ disease and mortality within 6 months after initiating cancer therapy. Comparison between the 3 groups was performed by Fischer exact test or Wilcoxon Rank sum as appropriate.

Characteristics of CMV infections

**Figure:**
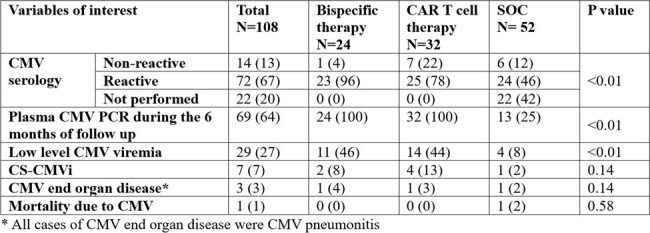

Abbreviations:

SOC: standard of care; CMV: cytomegalovirus; PCR: polymerase chain reaction; CS-CMVi: clinically significant CMV viremia

**Results:**

A total of 118 patients were screened; 6 did not receive any therapy of interest, 4 did not undergo follow up, therefore 108 patients were included in the analysis. Of those, 24, 32 and 52 R/R MM patients received BsAbs, CAR T cell and SOC therapy respectively. Patient characteristics are depicted in table 1. The rates of CMV viremia were higher in BsAb and CAR T cell recipients (46% and 44%) compared to SOC (8%). CS-CMVi was also higher in BsAb and CAR T cell recipients (8% and 13%) compared to SOC (2%) but this was not statistically significant (p=0.14) (Table 2). Three cases of CMV end organ disease were identified; all had CMV pneumonitis. There was one death due to CMV recorded.

**Conclusion:**

CMV viremia occurs more commonly amongst R/R MM undergoing treatment with T-cell redirecting therapies, such as BsAbs and CAR T cell in comparison with patients receiving SOC therapy. Further studies are needed to help determine the best strategies for CMV surveillance and viral load thresholds in these populations to mitigate the risk of CS-CMVi by early initiation of anti-CMV treatment.

**Disclosures:**

**Fareed Khawaja, MBBS**, Eurofins Viracor: Grant/Research Support|Symbio: Grant/Research Support **Krina Patel, MD**, Abbvie: Advisor/Consultant|Arcellx: Advisor/Consultant|Astra Zeneca: Advisor/Consultant|BMS: Advisor/Consultant|BMS: Scientific committee/chair|Caribou Sciences: Advisor/Consultant|Celgene: Advisor/Consultant|Genentech: Advisor/Consultant|Janssen: Advisor/Consultant|Kite: Advisor/Consultant|Kite: Scientific committee/chair|Merck: Advisor/Consultant|Oricel: Scientific committee/chair|Pfizer: Advisor/Consultant|Regeneron: Advisor/Consultant|Sanofi: Advisor/Consultant **Hans C. Lee, MD**, Abbvie: Advisor/Consultant|Allogene Therapeutics: Advisor/Consultant|Bristol Myers Squibb: Advisor/Consultant|Bristol Myers Squibb: Grant/Research Support|Genentech: Advisor/Consultant|GlaxoSmithKline: Advisor/Consultant|GlaxoSmithKline: Grant/Research Support|Janssen: Advisor/Consultant|Janssen: Grant/Research Support|Regeneneron: Advisor/Consultant|Regeneneron: Grant/Research Support|Sanofi: Advisor/Consultant|Takeda Pharmaceuticals: Advisor/Consultant|Takeda Pharmaceuticals: Grant/Research Support **Roy F. Chemaly, MD/MPH**, AiCuris: Advisor/Consultant|AiCuris: Grant/Research Support|Ansun Pharmaceuticals: Advisor/Consultant|Ansun Pharmaceuticals: Grant/Research Support|Astellas: Advisor/Consultant|Eurofins-Viracor: Grant/Research Support|InflaRX: Advisor/Consultant|Janssen: Advisor/Consultant|Karius: Advisor/Consultant|Karius: Grant/Research Support|Merck/MSD: Advisor/Consultant|Merck/MSD: Grant/Research Support|Moderna: Advisor/Consultant|Oxford Immunotec: Advisor/Consultant|Oxford Immunotec: Grant/Research Support|Roche/Genentech: Advisor/Consultant|Roche/Genentech: Grant/Research Support|Shinogi: Advisor/Consultant|Takeda: Advisor/Consultant|Takeda: Grant/Research Support|Tether: Advisor/Consultant

